# Generation and Characterization of a Transgenic Mouse Carrying a Functional Human ***β***-Globin Gene with the IVSI-6 Thalassemia Mutation

**DOI:** 10.1155/2015/687635

**Published:** 2015-05-04

**Authors:** Giulia Breveglieri, Irene Mancini, Nicoletta Bianchi, Ilaria Lampronti, Francesca Salvatori, Enrica Fabbri, Cristina Zuccato, Lucia C. Cosenza, Giulia Montagner, Monica Borgatti, Fiorella Altruda, Sharmila Fagoonee, Gianni Carandina, Michele Rubini, Vincenzo Aiello, Laura Breda, Stefano Rivella, Roberto Gambari, Alessia Finotti

**Affiliations:** ^1^Department of Life Sciences and Biotechnology, Biochemistry and Molecular Biology Section, Ferrara University, 44121 Ferrara, Italy; ^2^Laboratory for the Development of Pharmacological and Pharmacogenomic Therapy of Thalassemia, Biotechnology Center, Ferrara University, 44121 Ferrara, Italy; ^3^Department of Molecular Biotechnology and Health Sciences, Molecular Biotechnology Center, Turin University, 10126 Turin, Italy; ^4^Laboratory for Chemical and Clinical Analysis and Microbiology, University Hospital, Cona, 44124 Ferrara, Italy; ^5^Department of Biomedical and Specialty Surgical Sciences, Medical Genetic Unit, Ferrara University, 44121 Ferrara, Italy; ^6^Division of Hematology-Oncology, Department of Pediatrics, Weill Cornell Medical College, New York, NY 10065, USA

## Abstract

Mouse models that carry mutations causing thalassemia represent a suitable tool to test *in vivo* new mutation-specific therapeutic approaches. Transgenic mice carrying the *β*-globin IVSI-6 mutation (the most frequent in Middle-Eastern regions and recurrent in Italy and Greece) are, at present, not available. We report the production and characterization of a transgenic mouse line (TG-*β*-IVSI-6) carrying the IVSI-6 thalassemia point mutation within the human *β*-globin gene. In the TG-*β*-IVSI-6 mouse (a) the transgenic integration region is located in mouse chromosome 7; (b) the expression of the transgene is tissue specific; (c) as expected, normally spliced human *β*-globin mRNA is produced, giving rise to *β*-globin production and formation of a human-mouse tetrameric chimeric hemoglobin ^mu^ *α*-globin_2_/^hu^ *β*-globin_2_ and, more importantly, (d) the aberrant *β*-globin-IVSI-6 RNAs are present in blood cells. The TG-*β*-IVSI-6 mouse reproduces the molecular features of IVSI-6 *β*-thalassemia and might be used as an *in vivo* model to characterize the effects of antisense oligodeoxynucleotides targeting the cryptic sites responsible for the generation of aberrantly spliced *β*-globin RNA sequences, caused by the IVSI-6 mutation. These experiments are expected to be crucial for the development of a personalized therapy for *β*-thalassemia.

## 1. Introduction

In *β*-thalassemias, mutations of the *β*-globin gene or its regulatory regions cause absence (*β* ^0^) or reduced synthesis (*β* ^+^) of *β*-globin chains [1–4], associated with a corresponding excess of the complementary *α*-globins. The outcome of this unbalanced globin production is the destruction of erythroid precursors in bone marrow and at extramedullary sites (ineffective erythropoiesis) by apoptosis and short survival of red blood cells (RBCs) in the peripheral blood [5–9]. The disease is associated with morbidity and mortality due to severe chronic anemia or treatment-related complications.

More than 200 point mutations cause *β*-thalassemia [10] and can affect transcription, splicing of the primary transcript, translation, and stability of the *β*-globin mRNA. For instance, *β* ^0^39-thalassemia is caused by a stop codon mutation that leads to premature termination of *β*-globin chain synthesis [11, 12]; the *β* ^0^IVSI-1 mutation suppresses the correct maturation of the *β*-globin RNA precursor [13], while the *β* ^+^IVSI-110 allele coexpresses an abnormally spliced *β*-globin mRNA and a normal one [14].

Recently, the effort of several research groups has focused on the development of possible therapeutic interventions designed for patients carrying specific *β*-thalassemia mutations (personalized therapy). For instance, Salvatori et al. reported the proof-of-principle that aminoglycosides are able to restore to some extent HbA production in erythroid cells from homozygous *β* ^0^39-thalassemia patients [15]. Lonkar et al. described a PNA-based approach method for targeted correction of a thalassemia-associated *β*-globin mutation [16]. In addition, other groups approached a therapy based on the correction of aberrant pre-mRNA splicing [17, 18].

Mouse models for the different mutations causing thalassemia are, therefore, very important to test* in vivo* the activity of new potential approaches that target specific mutations [19]. The mouse *β*-globin locus contains four functional *β*-globin genes: *β*h1 and *ε* ^y^ (transcribed only during the embryonic phase of development and silenced in 14-15-day-old embryos) and the b1 (*β* ^major^) and b2 (*β* ^minor^) genes that are transcriptionally activated* in utero* around 11 days after conception [20]. Unlike in humans, *γ*-like globin genes are not present in mouse, and the embryonic to adult hemoglobin (Hb) switch occurs before birth (while in humans this switch occurs during the first 6 months after birth). Accordingly, mice homozygous for mutations that prevent expression of the *β*-globin genes die perinatally, due to the lack of expression of any Hb [19], although recently models mimicking *β* ^0^-thalassemia have been generated. These animals are viable at birth due to the prolonged expression of human fetal hemoglobin and then require chronic transfusions for survival [20, 21]. However, the most utilized adult murine models carry the complete deletion of one or both the mouse *β*-globin genes, showing phenotypic features similar to those observed in *β*-thalassemia intermedia patients [22, 23]. These animals do not carry any of the most common mutations observed in *β*-thalassemia in humans.

Therefore, murine models of *β*-thalassemia, which carry a mutated human *β*-globin gene in combination with the presence of deletions of the mouse *β*-like globin genes can be an invaluable tool to test new therapeutic strategies. For instance, Vadolas et al. generated a humanized mouse model carrying the common *β* ^+^IVSI-110 splicing mutation on a bacterial artificial chromosome including the human *β*-globin locus [24]. They examined heterozygous murine *β*-globin knock-out mice carrying either the IVSI-110 or the normal human *β*-globin locus. A 90% decrease in human *β*-globin chain synthesis in the IVSI-110 mouse model compared with the mouse model carrying the normal human *β*-globin locus was observed. This notable difference is attributed to aberrant splicing. The humanized IVSI-110 mouse model accurately mimics the splicing defect found in *β*-thalassemia patients with this mutation. This mouse model therefore offers a platform to test strategies for the restoration of normal splicing. Other examples of “humanized” transgenic mice proposed as model systems for *β*-thalassemia have been reported [25–27].

The generation of new transgenic mice carrying other specific *β*-thalassemia mutations might help the characterization and development of drugs that selectively target specific mutations. The IVSI-6 mutation is the most frequent in the Middle-Eastern region and is also recurrent in Italy and Greece [28–30]. This mutation leads to the activation of three cryptic splicing sites, which generate three aberrantly spliced mRNAs. The production of a mouse that expresses such mutation could supply a model to test new compounds and therapies for this population of patients. Therefore, we developed a novel and the first transgenic line carrying the human IVSI-6 *β*-globin gene.

## 2. Materials and Methods

### 2.1. Vector Design and Construction

For the production of transgenic mice, we designed a lentiviral vector containing the human *β*-globin gene under the control of its physiological promoter and a portion of the human locus control region (LCR), named pCCL.*β*-globin.PGK.GFP.WPRE (T9W) [31]. The vector T9W-IVSI-6 was generated by* in vitro* mutagenesis, introducing the IVSI-6 *β*-thalassemic point mutation inside the human *β*-globin gene. Mutagenesis has been performed by using the QuickChange II Site-Directed Mutagenesis Kit (Stratagene, La Jolla, CA, USA) [32]. A double stranded mutant oligonucleotide (5′-CCTGGGCAGGTTGG**C**ATCAAGGTTACAAG-3′) was used in order to introduce the IVSI-6 mutation into the *β*-globin gene. The mutagenesis reaction has been performed in a final volume of 25 *μ*L, containing 25 ng of plasmid template, 1x Reaction Buffer (20 mM Tris-HCl pH 8.8, 2 mM MgSO_4_, 10 mM KCl, 10 mM (NH_4_)_2_SO_4_, 0.1 mg/mL BSA, 0.1% Triton X-100), 0.5 *μ*L of dNTP Mix, 62.5 ng of mutagenesis primers, by using 1.25 U of PfuUltra HF DNA polymerase. The thermal reaction has been performed by using the GeneAmp PCR System 9600 (Perkin Elmer, Waltham, MA, USA): after a first denaturation at 94°C for 3 minutes, 22 cycles were performed, consisting of denaturation at 95°C for 30 seconds, annealing at 55°C for 1 minute and elongation at 68°C for 8 minutes. At the end of the mutagenesis reaction, the amplification product was digested with 5 U of the restriction endonuclease DpnI, at 37°C for 1 hour, so as to remove the parental not mutated DNA. 5 *μ*L of the digestion reaction was then used to transform 120 *μ*L of ultracompetent* E. coli* JM109 bacteria: DNA and bacteria were incubated on ice for 4 hours and, then, after a thermic shock at 42°C for 45 seconds and immediately on ice for 2 minutes, 1 mL of Luria Bertani Medium (LB Medium: 10 g/L bacto-tryptone, 5 g/L yeast extract, 10 g/L NaCl) was added and an incubation at 37°C for 1 hour under slow agitation was performed; finally bacteria have been plated on Petri plates containing semisolid medium (LB Medium with 15 g/L bacto-agar) in the presence of 100 *μ*g/mL ampicillin and incubated at 37°C for one night. The bacterial clones obtained were screened for the incorporation of the recombinant plasmid construct, whose nucleotide sequence was finally confirmed by DNA sequencing.

### 2.2. Production of Transgenic Mice by Microinjection

The 6.1 Kb XcmI-ClaI fragment corresponding to the *β* ^+^IVSI-6 insert was purified with the QIAquick Gel Extraction Kit (QIAGEN, Hilden, Germany) according to manufacturer's instructions and sterile filtered with a 0.22 *μ*m Costar Spin-X column (Corning Incorporated, Corning, NY, USA). Five hundred DNA molecules/picoliter were microinjected in the pronucleus of fertilized eggs of 8-week-old FVB mice. The injected embryos were implanted into CBA/J X C57BL/6J pseudopregnant females and the offspring genotype was tested for the integration of the transgene as described below.

### 2.3. Transgenic Mice

Mouse strains were supplied by Molecular Biotechnology Center of Turin University. Maintaining and experimental procedures were done at Ferrara University with the approval of Ethics Committee.

### 2.4. Purification of Murine Genomic DNA

Murine genomic DNA was purified from mouse tails. Briefly, 1x DreamTaq Buffer (containing KCl, (NH_4_)_2_SO_4_, 20 mM MgCl_2_) (Fermentas, Burlington, ON, Canada) and 0.2 mg/mL proteinase K were added to a 0.2–0.5 cm tail snip in a final volume of 50 *μ*L, before incubating at 57°C in a water bath for 16–20 hours. The samples were briefly vortexed and incubated at 95°C for 10 minutes to inactivate proteinase K and, finally, after centrifuging at maximum speed for 5 minutes, the supernatant containing genomic DNA was collected. Purified genomic DNA was checked by 0.8% agarose gel electrophoresis and quantified by spectrophotometry.

### 2.5. Synthetic Oligonucleotides

The nucleotide sequences of PCR primers were designed using the Primer Express Oligonucleotide Selection Software, version 1.0 (Applied Biosystems, Life Technologies, Carlsbad, CA, USA) and are reported in Tables 1 and 2. HPLC-grade oligonucleotides were purchased from Sigma Genosys (Cambridge, UK).

### 2.6. Polymerase Chain Reaction (PCR)

In each PCR reaction, 1 *μ*L of murine genomic DNA was amplified by DreamTaq DNA polymerase (Fermentas): PCR was performed in a final volume of 100 *μ*L, containing 1x DreamTaq Buffer (containing KCl, (NH_4_)_2_SO_4_, 20 mM MgCl_2_), 33 *μ*M dNTPs, 150 ng of PCR primers, and 1.25 U of DreamTaq DNA polymerase. PCR primer pairs used (Table 1) were as follows:* MuActF* (forward) and* MuActR* (reverse), designed to amplify a 871 bp sequence located on the murine *β*-actin gene;* TransF* (forward) and* TransR* (reverse), which amplify a 154 bp sequence on the transgene;* HuBetaF* (forward) and* HuBetaR* (reverse), designed to amplify a 449 bp sequence on the human *β*-globin gene. The amplification cycles used were as follows: denaturation, 30 sec, 95°C; annealing, 20 sec, temperature 1-2°C lower than primer melting temperatures; elongation, 72°C for a length of time depending on the PCR product size.

### 2.7. Sequencing of PCR Products

*HuBetaF-HuBetaR* PCR products, containing part of the human *β*-globin gene, were purified with MicroCLEAN (Microzone Limited, Haywards Heath, West Sussex, UK) and sequenced by using the ABI PRISM BigDye Terminator Cycle Sequencing Ready Reaction Kit, v1.0 (Applied Biosystems). Sequence reactions were performed in a final volume of 20 *μ*L, containing 40 ng of PCR template, 3.2 pmoles of primer* HuBetaR*, 1x Sequencing Buffer, and 8 *μ*L of Terminator Ready Reaction Mix. 45 amplification cycles were performed, as follows: denaturation, 96°C, 10 seconds; annealing, 65°C, 5 seconds; elongation, 65°C, 3 minutes. A denaturing 4% polyacrylamide gel electrophoresis was then carried out in an automated ABI PRISM 377 DNA Sequencer (Applied Biosystems), and final sequence data were analyzed by Sequencing Analysis 3.3 (Applied Biosystems) and Chromas Lite 2.01 (Copyright^©^ 2003–2008 Technelysium Pty Ltd.) softwares.

### 2.8. Quantification of Human *β*-Globin Genes in Transgenic Mice by Real-Time PCR

Calibration curves were obtained using 50, 100, and 150 ng of genomic DNA from a hemizygous mouse and the *β*-actin gene as endogenous control. The relative *β*-globin/actin gene ratio in investigated mice was compared to the same ratio in the hemizygous control mouse. Quantitative real-time PCR assay was carried out using gene-specific double fluorescently labeled probes. The primers and probes used for real-time PCR analysis of human *β*-globin gene (Assay ID Hs00758889_s1) and of mouse cytoplasmic *β*-actin (Assay ID Mm00607939_s1) were purchased from Applied Biosystems. The hemizygous or homozygous status of transgenic mice was determined by relative real-time PCR, taking a hemizygous DNA as a reference, by using the comparative cycle threshold method [15, 33, 34].

### 2.9. Quantitative Multiplex PCR of Short Fluorescent Fragments (QMPSF)

To determine transgene dosage comparing and discriminating homozygous from hemizygous samples, dosage quotients (DQ) were obtained by QMPSF assays as reported by Yau et al. [35] and Feriotto et al. [36]. A 2-fragment multiplex PCR assay was performed to amplify a 154 bp transgene sequence, using primers* TransF[6FAM]* and* TransR* (Table 1) and a 201 bp fragment belonging to the murine *β*-actin gene, used as a normalization control, by using primers* MuActF1[6FAM] *and* MuActR1* (Table 1). All forward primers in the assay were 5′-labeled with the fluorescent phosphoramidite 6-FAM (Sigma Genosys).

Amplifications were performed in 25 *μ*L volumes, containing 125 ng genomic DNA, 0.01–0.02 *μ*M forward primers (unlabeled reverse primers were used as 1.4-fold excess respect to the corresponding forward primers; relative ratios between transgene primers and *β*-actin primers were 0.3 : 0.6), 66 *μ*M dNTPs, and 0.7 U of DreamTaq DNA polymerase (Fermentas). After 6-minute initial denaturation at 96°C, a “hot start” amplification was initiated by adding DreamTaq DNA polymerase, followed by 19 cycles consisting of a 15 seconds denaturation step at 95°C, a 30 seconds annealing step at 64°C, and a 15 seconds extension step at 72°C, with a final extension for 45 minutes at 72°C. The PCR products were analyzed by electrophoresis and the fluorescent signals were identified by using the ABI GeneScan Analysis Software, version 3.1 (Applied Biosystems) to produce electropherograms in which areas under the peaks represent the amount of PCR products. The molecular weight marker used was the GeneScan 400HD [Rox] Dye Size Standard (Applied Biosystems), designed for sizing DNA fragments in the 50–400 nucleotides range. In order to determine transgene dosage and to compare and discriminate homozygous and hemizygous samples, dosage quotients (DQ) were obtained as elsewhere described [35, 36].

### 2.10. Hematological Analysis

Blood was collected from 16-week-old transgenic mice by retroorbital bleeding into tubes containing EDTA and analyzed by an automated Sysmex XE 2100 hematological analyzer (TOA Sysmex, Japan) at the Laboratory for Chemical and Clinical Analysis and Microbiology, University Hospital, Ferrara, Italy.

### 2.11. Fluorescence* In Situ* Hybridization (FISH) Analysis

Fibroblast cell cultures were established in DMEM medium (Gibco, Life Technologies, Carlsbad, CA, USA) with nonessential aminoacids (Sigma-Aldrich, St. Louis, MO, USA), penicillin/streptomycin and 10% fetal calf serum, from tail samples from transgenic mice. The cells were grown for 10–14 days and then harvested following colcemid inhibition of cell division for 3–6 h. Chromosome preparations were obtained by using standard techniques. A probe was prepared from the intact T9W-IVSI-6 vector, directly labeled by nick translation with the DIG-Nick Translation Mix (Roche Applied Science, Penzberg, Upper Bavaria, Germany) according to the manufacturer's protocol. The probe was hybridized and then detected with anti-digoxigenin-fluorescein Fab fragments (Roche Applied Science). The slides were mounted in Vectashield (Vector Laboratories, Burlingame, CA) containing 4′,6-diamidino-2-phenylindole (DAPI) counterstain. FISH signals were examined with Olympus Provis epifluorescence microscope and images were captured using Leica Microsystems CytoVision imaging equipment and software (Applied Imaging, Leica-Microsystems, Wetzlar, Germany). The chromosomal site of transgene integration was determined by karyotypic analysis of banded chromosomes obtained using the DAPI image.

### 2.12. RT-PCR

Total RNA was obtained from 250 *μ*L of wild-type, hemizygous, and homozygous mouse whole blood using the Mouse RiboPure Blood RNA Isolation Kit (Ambion/Applied Biosystems, Austin, TX, USA). cDNA was synthesized from 250 ng of total RNA using Superscript VILO cDNA Synthesis kit (Invitrogen, Carlsbad, CA, USA). PCR was performed using a GeneAmp PCR 9700 System (Perkin Elmer, Waltham, MA, USA), 1/20 of reverse transcription reaction mixture (cDNA), 125 ng of human genomic DNA or 20 ng of pCCL.*β*-globin.PGK.GFP.WPRE (T9W) [31] vector DNA, 2 U of DyNAzyme DNA polymerase (Finnzymes, Oy, Espoo, FI), and 33 *μ*M deoxynucleoside triphosphates. The* HuBetaF1* forward and the* HuBetaR* reverse primers (Table 2) were designed to amplify a 153 bp fragment of the human *β*-globin transcript or a 283 bp product of the corresponding human genomic DNA. The* MuBetaF* forward and* MuBetaR* reverse primers (Table 2) were used to amplify a 147 bp product of the mouse *β*-globin transcript. The PCR conditions were as follows: 35 cycles of amplification, which included a 20-second denaturation step at 95°C, a 30-second annealing step at 66°C, and a 25-second elongation step at 72°C.

### 2.13. Real-Time RT-PCR

For RNA extraction, transgenic mouse tissues were homogenized using IKA T10 Basic Ultraturrax (IKA Werke GmbH & Co. KG, Staufen, DE) directly in TRIzol Reagent (Invitrogen) and 1 *μ*g of the obtained total RNA was treated with RQ1 DNAse (Promega, Madison, WI, USA) to remove genomic DNA contaminations. cDNAs were synthesized from 250 ng of total RNA using Superscript VILO cDNA Synthesis kit (Invitrogen). For quantitative real-time PCR reaction, 0.8/20 *μ*L of cDNA, 150 ng of each primer (Table 2), and 1x iQ SYBR Green Supermix (Bio-Rad, Hercules, CA, USA) were used for each reaction.* HuBetaF1* and* HuBetaR* primers were designed to amplify a 153 bp sequence present in transgenic human *β*-globin transcripts,* MuBetaF-MuBetaR* primers and* MuAlphaF-MuAlphaR* primers were designed to amplify a 147 bp sequence present in mouse *β*-globin transcript and a 306 bp sequence present in mouse *α*-globin transcript, respectively, while* MuActF2* and* MuActR2* primers were designed to amplify a 331 bp sequence of mouse *β*-actin transcript (Table 2). Primer pairs and amplification conditions were validated by melting curve and electrophoretic analysis. Real-time PCR reactions were performed for a total of 40 cycles (95°C for 10 s, 66°C for 30 s, and 72°C for 25 s) using an iCycler IQ5 (Bio-Rad). The relative proportions of each template amplified were determined by using the IQ5 software (Bio-Rad), employing the ΔΔCt method [15, 33, 34] to compare gene expression data.

### 2.14. Cell Lines and Culture Conditions

Murine erythroleukemia (MEL) cells [37] were grown in modified Dulbecco's minimal essential medium (D-MEM, Lonza Group, Basel, CH) supplemented with 10% fetal bovine serum (BioWest, Nuaillé, France), penicillin (550 units/mL), and streptomycin (75 units/mL) (Lonza Group) at 37°C in 5% CO_2_ humidified atmosphere. Cell growth was monitored daily using a Burker chamber. Cell viability was measured by trypan blue staining (Burr, BDH Chemicals, Poole, England) [38].

### 2.15. *In Vitro* Culture of Erythroid Progenitors from IVSI-6 *β*-Thalassemia Patients

Blood samples from healthy donors and homozygous IVSI-6 patients were collected after receiving informed consent. The two-phase liquid culture procedure was employed as previously described [39, 40]. Mononuclear cells were isolated from peripheral blood samples of normal donors by Ficoll-Hypaque density gradient centrifugation and seeded in *α*-minimal essential medium (*α*-MEM, Sigma Genosys) supplemented with 10% FBS (Celbio, Milano, Italy), 1 *μ*g/mL cyclosporine A (Sandoz, Basel, Switzerland), and 10% conditioned medium from the 5637 bladder carcinoma cell line. The cultures were incubated at 37°C, under an atmosphere of 5% CO_2_. After 7 days in this phase I culture, the nonadherent cells were harvested, washed, and then cultured in phase II medium, composed of *α*-MEM (Sigma Genosys), 30% FBS (Celbio), 1% deionized bovine serum albumin (BSA, Sigma Genosys), 10^−5^ M *β*-mercaptoethanol (Sigma Genosys), 2 mM L-glutamine (Sigma Genosys), 10^−6^ M dexamethasone (Sigma Genosys), and 1 U/mL human recombinant erythropoietin (EPO) (Tebu-bio, Magenta, Milano, Italy), and stem cell factor (SCF, BioSource International, Camarillo, CA, USA) at the final concentration of 10 ng/mL. Erythroid differentiation was assessed by benzidine staining, in a solution containing 0.2% benzidine HCl (Sigma Genosys) in 0.5 M glacial acetic acid, preactivated with 10% (v/v) of a solution 30% H_2_O_2_ [36].

### 2.16. Induction of Erythroid Differentiation and Transduction of MEL Cells

MEL cells were stimulated to differentiation by dimethyl sulfoxide (DMSO) [37] (Sigma Genosys) and transduced with the lentiviral vectors T9W [31] or T9W-IVSI-6. The infection was performed by plating 2 × 10^6^ MEL cells in 3 mL of medium in a 6-well plate; then fresh 2% v/v DMSO was added and cells were incubated 18–20 hours at 37°C in a humidified incubator in an atmosphere of 5% CO_2_. Then, MEL cells were infected with T9W, a lentiviral vector carrying the human *β*-globin gene and large elements from the human locus control region (LCR), at an MOI (multiplicity of infection) of 0.5. We used polybrene at 8 *μ*g/mL final concentration to facilitate viral entry and then incubated the cells for 16 hours at 37°C in a humidified incubator in an atmosphere of 5% CO_2_. The infected cells were collected by centrifuging at 300 g for 5 minutes at room temperature, to remove the medium containing not integrated viral particles. The cells were then resuspended in 3 mL of fresh medium and replated in a 6-well plate. After 10 hours, MEL cells were collected and spun at 300 g for 5 minutes at room temperature. The treatment of MEL cells with T9W-IVSI-6 vector was performed as just described, after plating 5 × 10^5^ MEL cells in 1 mL of medium in a 24-well plate. Cells were counted in a Burker chamber and the benzidine positive ones were determined as percentage as elsewhere reported [36].

### 2.17. Western Blotting

10 *μ*L of 1 : 200 diluted mouse whole blood was analyzed and 4 *μ*g of human adult hemoglobin A_0_ (H-0267, Sigma Genosys) was used as migration reference; proteins were denatured for 5 minutes at 98°C in SDS gel loading buffer 1x (50 mM Tris-HCl pH 6.8, 2% SDS, 100 mM Dithiothreitol (DTT), 0.1% bromophenol blue, 10% glycerol) and separated by SDS-PAGE, by using a 10 cm × 8 cm gel and Tris-glycine Buffer (25 mM Tris, 192 mM glycine, 0.1% SDS). The electrotransfer to 20 microns nitrocellulose membrane was performed for 3 hours at 400 mA and 4°C, in electrotransfer buffer (25 mM Tris, 192 mM glycine, 5% methanol). The membrane was prestained in Ponceau S Solution (Sigma Genosys) to verify the transfer, washed with 25 mL Tris-buffered saline (TBS) (10 mM Tris-HCl pH 7.4, 150 mM NaCl) for 10 minutes at room temperature and incubated in 20 mL of blocking buffer (TBS, 0.1% Tween-20, 5% w/v nonfat dry milk) for 1 hour at room temperature. The membrane was then incubated with primary mouse monoclonal antibody (1 : 200) (sc-21757, Santa Cruz Biotechnology, Santa Cruz, CA, USA) targeting the human *β*-globin, in 10 mL of blocking buffer with gentle agitation overnight at 4°C. The day after, the membrane was washed three times for 5 minutes each with 20 mL of TBS/T (TBS, 0.1% Tween-20) and incubated with 25 ng/mL anti-mouse HRP-conjugated secondary antibody (1 : 2000) (Pierce Thermo Scientific, Rockford, IL, USA) in 10 mL TBS/T with gentle agitation for 1 hour at room temperature. After three washes, each with 15 mL of TBS/T for 5 minutes, finally the membrane was incubated with 5 mL of Western Lightning Chemiluminescence Reagent Plus (PerkinElmer Sciences, Waltham, MA, USA) with gentle agitation for 1 minute at room temperature and exposed to X-ray film (Amersham Hyperfilm ECL, GE Healthcare, Buckinghamshire, UK). For western blotting in nondenaturing conditions, 10 *μ*L of 1 : 200 diluted mouse whole blood, 30 *μ*g of MEL cells extracts, and 500 ng of human adult hemoglobin A_0_ (H-0267, Sigma Genosys) were diluted in 1x native gel loading buffer (50 mM Tris-HCl pH 8.8, 0.1% bromophenol blue, 10% glycerol) and separated by a NATIVE-PAGE, by using a 10 cm × 8 cm gel and Tris-glycine buffer without SDS. The following steps and conditions were the same described above for denaturing western blotting.

### 2.18. Capillary Electrophoresis (CE)

High voltage CE was performed by using the Minicap Flex Piercing capillary system (Sebia, Lisses, France). Manufacturer's guidelines were followed in performing the analysis. Sample processing required a 1 : 6 dilution of 50 *μ*L whole blood with hemolysing solution and vortexing for 5 seconds. After loading the primary sample tubes into the carousel, the instrument performed automated bar code reading, mixing of the samples by inversion, cap piercing, sampling, and dilution. Electrophoresis was performed at alkaline pH (9.4), high voltage (9500 V), and controlled temperature. The hemoglobin bands were detected by absorption photometry, and optical density measurements were converted to a migration image, displayed as a graph called “electropherogram.” The migration position is measured in arbitrary units between 0 and 300 and can be quantified as a percentage. Results were acquired and examined by using the Sebia Phoresis REL 8.6.2 Software.

### 2.19. RT-PCR for Alternatively Spliced Transcripts

After RQ1 DNAse (Promega) treatment, 250–500 ng of total RNA were used to synthesize cDNAs, using Superscript VILO cDNA Synthesis kit (Invitrogen), according to the manufacturer's instructions. PCR was performed using 2 out of 20 *μ*L of reverse transcription reaction mixture, 2 U of DyNAzyme DNA polymerase (Finnzymes) and 33 *μ*M deoxynucleoside triphosphates. Fluorescent PCR products were obtained using the* HuBetaF1[6-FAM]* forward and* HuBetaR *reverse primers (Table 2) and detected after electrophoresis in a denaturant polyacrylamide gel using the ABI GeneScan Analysis Software, version 3.1 (Applied Biosystems). For the specific amplification of the aberrant transcript caused by the activation of the cryptic splicing site at position IVSI+13,* IVSI+13F* forward and* HuBetaR* reverse primers (Table 2), designed to amplify, after retrotranscription, 84 bp of human *β*-globin alternatively spliced transcript or 202 bp of human pre-mRNA (containing all the first human *β*-globin intron), were employed. The PCR conditions were as follows: 40–50 cycles of amplification, with 10 seconds of denaturation at 95°C, 30 seconds of annealing at 66°C, and 15 seconds of elongation at 72°C. Negative controls (no template cDNA) were also run to assess specificity and to rule out contamination.

## 3. Results

### 3.1. Vector Design and Construction

**Figure 1:**
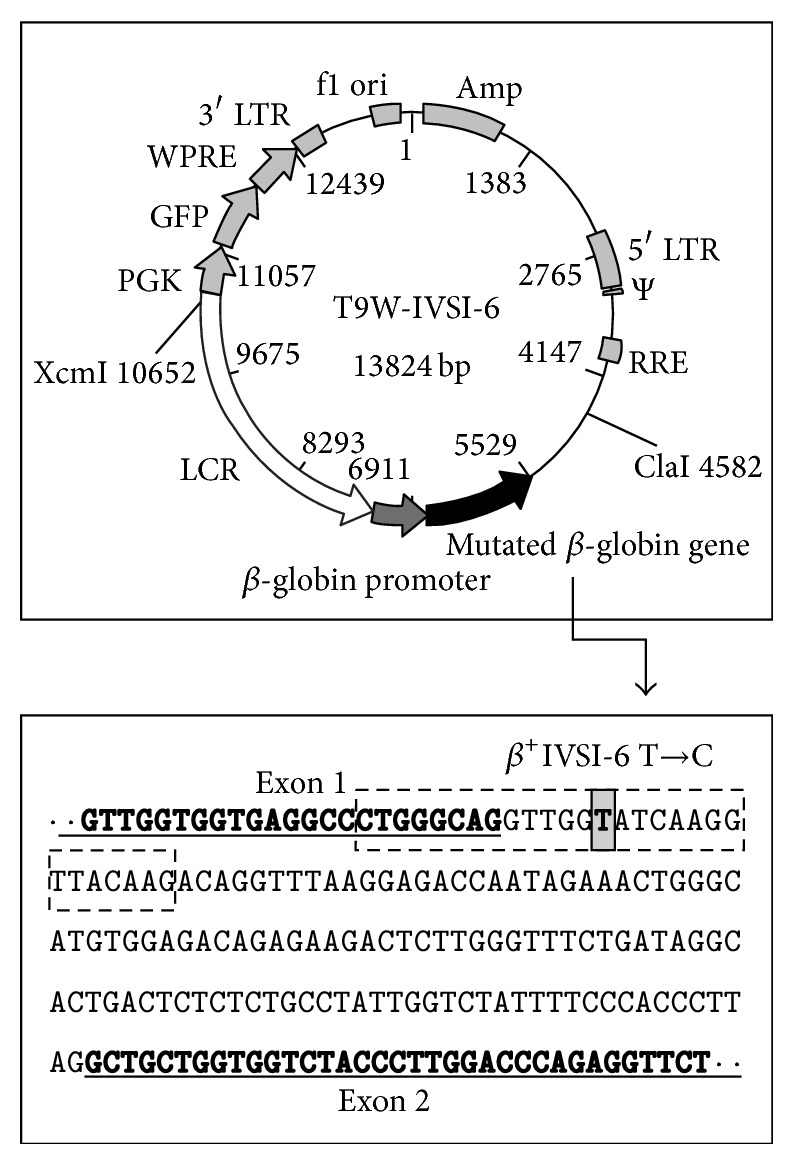
Map of the T9W-IVSI-6 vector showing the XcmI and ClaI restriction sites, used to cut the vector for microinjection. The human *β*-globin genomic region containing the *β* ^+^IVSI-6 thalassemic point mutation (grey box) is also reported. The final portion of the *β*-globin exon 1 and the first portion of the *β*-globin exon 2 are underlined and marked in bold characters. The position of mutagenesis primers is boxed with a dashed line.

For the generation of transgenic mice, we designed and produced a construct modifying the pCCL.*β*-globin.PGK.GFP.WPRE (T9W) cassette previously described [31]. This cassette contains the human *β*-globin gene under the control of its physiological promoter and a portion of the human locus control region (LCR) (Figure 1). The construct, named T9W-IVSI-6, was generated by* in vitro* mutagenesis, by introducing the *β* ^+^IVSI-6 point mutation, one of the most common molecular defects present in the *β*-thalassemia populations of Italy and Greece, in the human *β*-globin gene.

### 3.2. Production of the Transgenic Founder Mouse Carrying the Human *β*-Globin Gene with the *β* ^+^IVSI-6 Mutation (TG-*β*-IVSI-6)

Potential TG-*β*-IVSI-6 founder mice were produced by microinjection of the purified 6.1 Kb XcmI-ClaI fragment, corresponding to the *β* ^+^IVSI-6 insert, from the construct T9W-IVSI-6 (Figure 1).

**Figure 2:**
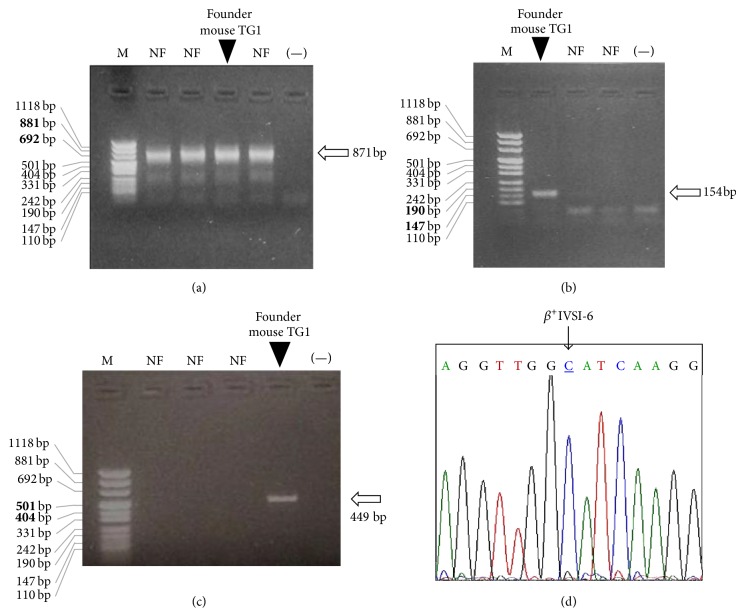
Identification of the founder mouse of the transgenic *β* ^+^IVSI-6 line. (a–c) Electrophoretic analyses of PCR products obtained by the amplification of murine genomic DNAs with primers* MuActF-MuActR* (a),* TransF-TransR* (b), and* HuBetaF-HuBetaR* (c), recognizing the murine *β*-actin gene, the transgene sequence, and the human *β*-globin gene, respectively. NF, negative founders; (−), negative control (water added to the amplification mixture); M, molecular weight ladder, pUC Mix Marker 8 (Fermentas). Arrows indicate the expected position of the specific amplification products; the lanes carrying the specific PCR products, relative to the founder mouse TG1, are also indicated. (d) Portion of electropherogram obtained by sequencing the* HuBetaF-HuBetaR* PCR product, obtained by the founder mouse TG1. The arrow indicates the peak corresponding to the *β* ^+^IVSI-6 thalassemic point mutation (underlined nucleotide).

For the screening and identification of the transgenic founders, murine genomic DNA was purified from the tails and analyzed by polymerase chain reaction (PCR).  Figure 2(b) shows that only the genomic DNA of the TG-*β*-IVSI-6 founder mouse (founder mouse TG1) was amplified by using* TransF* and* TransR* primers (Table 1), which anneal to the transgene sequence, while all the analyzed samples were amplified by using PCR primers specific for the murine *β*-actin gene (Figure 2(a)). Accordingly, Figure 2(c) shows the electrophoretic analysis of PCR products obtained by the amplification of four samples of murine genomic DNA with primers* HuBetaF* and* HuBetaR* (Table 1), specific for the human *β*-globin gene: again, the expected 449 bp band was generated only by the amplification of genomic DNA belonging to the founder mouse TG1. The 449 bp PCR product shown in Figure 2(c) was sequenced to confirm that the *β* ^+^IVSI-6 thalassemic point mutation was present in the DNA of the TG-*β*-IVSI-6 founder mouse (Figure 2(d)).

### 3.3. Characterization of the TG-*β*-IVSI-6 Homozygous Mice

**Figure 3:**
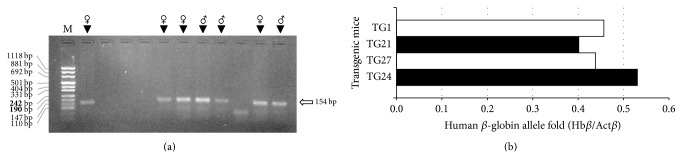
Identification of hemizygous mice among animals belonging to the F1 generation. (a) Agarose gel electrophoretic analysis of PCR products obtained by the amplification of genomic DNA purified from eleven mice with primers* TransF* and* TransR*, recognizing the transgene sequence. The arrow indicates the position of the expected 154 bp product; the sex of mice having generated a specific amplification band is also shown. M, molecular weight ladder, pUC Mix Marker 8 (Fermentas). (b) Human *β*-globin allele quantification by real-time PCR using primers and probes specific for human *β*-globin (Hb*β*) gene and mouse cytoplasmic *β*-actin (Act*β*) gene. Results of analysis of transgenic hemizygous mice TG21, TG24, and TG27, together with the founder mouse TG1, are reported as fold of human *β*-globin allele amount quantified with respect to the murine *β*-actin gene.

As a first step to produce homozygous *β* ^+^IVSI-6 transgenic lines, the founder mouse was back-crossed with wild-type mice.  Figure 3(a) shows the electrophoretic migration of PCR products obtained by the amplification of genomic DNA purified from eleven mice belonging to the F1 generation, with primers recognizing the transgene sequence. The arrow indicates the position of the 154 bp expected PCR product of the human *β*-globin transgene: we obtained three male and four female hemizygous mice. Homozygous animals were finally produced by crossing hemizygous F1 mice. In order to get preliminary information on the number of integration events occurring, quantitative real-time PCR assays were carried out by using gene-specific double fluorescently labeled probes. The analyses of three hemizygous mice (TG21, TG27 and TG24) are shown in Figure 3(b) and show a *β*-globin/*β*-actin ratio of about 0.5, compatible with a single copy integration of the transgene.

**Figure 4:**
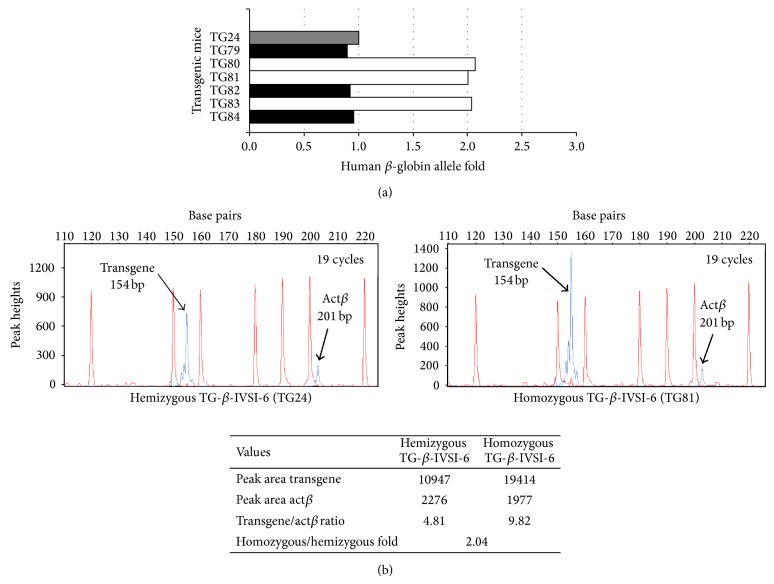
Discrimination between hemizygous and homozygous genotypes of transgenic mice by quantitative real-time PCR (a) or quantitative multiplex PCR of short fluorescent fragments (QMPSF) (b). (a) The amount of human *β*-globin allele was calculated using the comparative cycle threshold method [15, 33, 34] employing transgenic mouse TG24 as one copy control (hemizygous reference, grey). The black and white histograms represent hemizygous or homozygous transgenic (TG) mice, respectively. (b) Electropherograms obtained after denaturing polyacrylamide gel electrophoresis of amplification products of multiplex PCRs: primers employed (Table 1) were* TransF[6FAM]-TransR* and* MuActF1[6FAM]-MuActR1*, recognizing the transgene and the murine *β*-actin gene (Act*β*), respectively; templates were genomic DNAs purified from a hemizygous (upper left panel) or a homozygous (upper right panel) mouse. Peaks generated by the molecular weight ladder (120, 150, 160, 180, 190, 200, 220 bp fragments) and by the amplification products (transgene and Act*β*, indicated by the arrows) are reported in red and blue, respectively. Values obtained, as peak areas, transgene/Act*β* ratio and fold of ratios calculated from homozygous and hemizygous animals are shown in the lower part of panel (b).

As a second step, in order to discriminate hemizygous and homozygous mice, real-time PCR analyses were performed.  Figure 4(a) shows the real-time PCR analysis of six F2 transgenic mice, three of which are hemizygous (TG79, TG82, and TG84) and three homozygous (TG80, TG81, and TG83), according to the *β*-globin gene amplification compared to the F1 hemizygous TG24 mouse. These data were confirmed by quantitative multiplex PCR of short fluorescent fragments (QMPSF), as indicated by the representative example shown in Figure 4(b), performed on TG24 and TG81 mice. In the lower panel of Figure 4(b), the absolute value of human *β*-globin (transgene) and mouse *β*-actin (Act*β*) peak areas are indicated, together with their relative ratio in hemizygous and homozygous mice. These data indicate the establishment of the homozygous line.

### 3.4. Chromosomal Localization of the Human *β*-IVSI-6 Transgene

**Figure 5:**
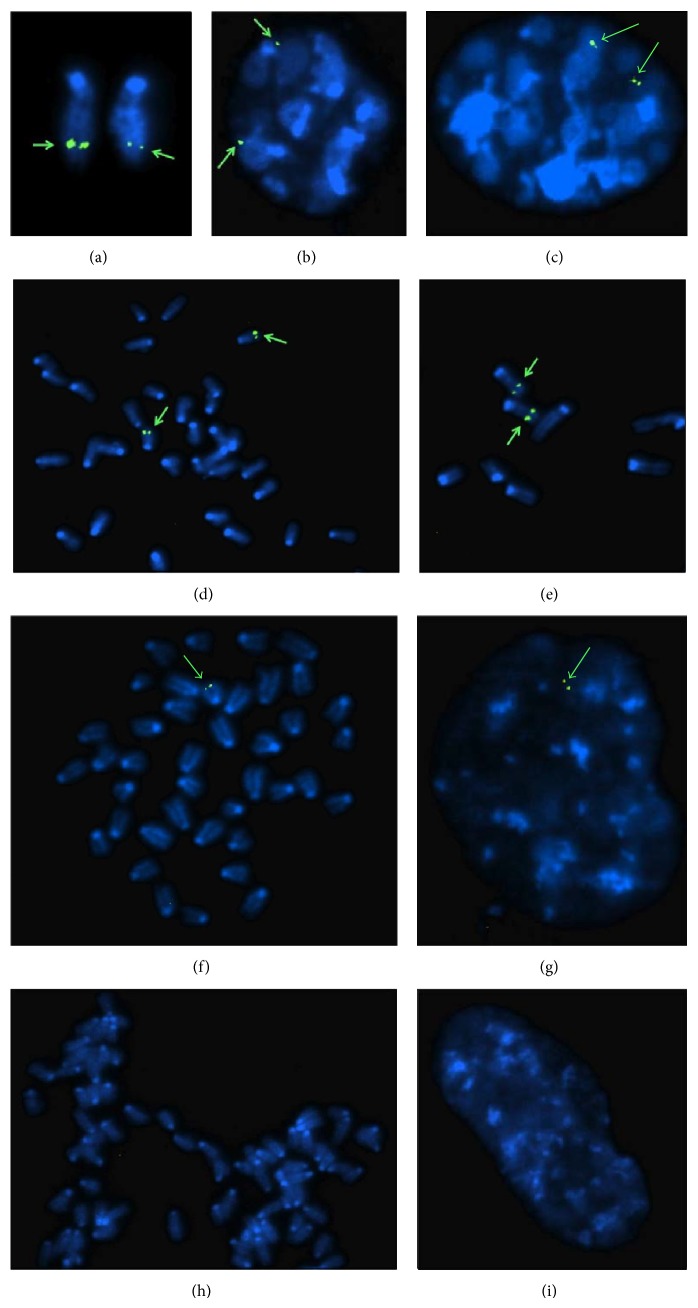
Fluorescence* in situ* hybridization results on metaphase (a, d, e, f, h) or G1 (b) and G2 (c, g, i) interphase nuclei of homozygous (a–e), hemizygous (f-g), and wild-type mice (h-i). The arrows indicate the integration site of the transgene, located in mouse chromosome 7.

Figure 5 shows representative FISH analyses performed on wild-type, hemizygous, and homozygous *β* ^+^IVSI-6 transgenic mice, demonstrating that integration occurred at band F2 of the mouse chromosome 7. As clearly shown, no FISH signals were found in wild-type samples (Figures 5(h) and 5(i)). Only one chromosome 7 gave FISH signals in hemizygous samples (Figures 5(f) and 5(g)), while in homozygous samples both chromosomes gave FISH signals (Figures 5(a)–5(e)). These data support the concept that only one integration unit of the human *β* ^+^IVSI-6 transgene is present in the produced homozygous *β* ^+^IVSI-6 transgenic mice. These data have been reproduced several times obtaining identical results.

### 3.5. Tissue Specific Expression of the Human *β*-IVSI-6 Transgene

**Figure 6:**
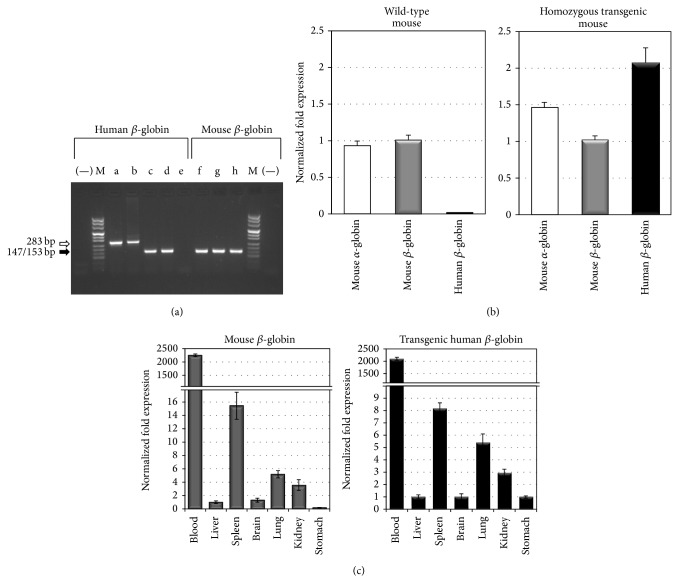
Expression of human *β*-globin transcripts in the transgenic TG-*β*-IVSI-6 mouse model. (a) RT-PCR was performed with total RNA isolated from wild-type (lanes e and h), transgenic hemizygous (lanes c and f), and transgenic homozygous (lanes d and g) mice with primers* HuBetaF1* and* HuBetaR* (Table 2) designed to specifically amplify a 153 bp fragment of human *β*-globin transcripts (lanes a–e) and primers* MuBetaF* and* MuBetaR* (lanes f–h) designed to specifically amplify a 147 bp fragment of mouse *β*-globin transcripts (black arrow). Genomic DNA and pCCL.*β*-globin.PGK.GFP.WPRE vector DNA were also used as control templates (lanes a and b, 283 bp, white arrow). M, molecular weight ladder, pUC Mix Marker 8 (Fermentas), (−), negative control for each primer pair. (b) SYBR Green real-time PCR was used to determine the relative expression of mouse *α*-globin, mouse *β*-globin, and human *β*-globin transcripts in wild-type mouse blood (left side of the panel) and in homozygous transgenic mouse blood (right side of the panel). (c) Relative expression levels of mouse *β*-globin (grey bars) and human *β*-globin (black bars) transcripts in transgenic mouse tissues using real-time RT-PCR. Mean ± SD values were determined for each fold difference; the relative proportions of *β*-globin/*β*-actin in each template were determined by using IQ5 software (Bio-Rad), employing the ΔΔCt method [15, 33, 34].

Figure 6(a) shows the RT-PCR analysis performed with total RNA isolated from wild-type (lanes e and h), transgenic hemizygous (lanes c and f) and transgenic homozygous (lanes d and g) TG-*β*-IVSI-6 mice using* HuBetaF1* and* HuBetaR* primers (lanes c, d, e), which selectively amplify human *β*-globin transcript, and primers* MuBetaF* and* MuBetaR* (lanes f, g, h) specific for mouse *β*-globin transcript. All the samples, amplified using the murine specific primers, generated the expected 147 bp product, whereas the human *β*-globin PCR product (153 bp) was obtained only from transgenic animals, but not from wild-type mice. Genomic DNA and T9W vector DNA were also amplified with* HuBetaF1* and* HuBetaR* primer pair (lanes a and b), showing a 283 bp product containing the intronic sequence as well.

The analysis confirms the human *β*-IVSI-6 transgene expression and the quantitative RT-PCR analyses shown in Figure 6(b) support this evidence: amplification employing the* HuBetaF1* and* HuBetaR* primers (black bar) was indeed observed only in the blood of transgenic mice. No significant differences were found in the endogenous *α* and *β*-globin expression between wild-type and transgenic mouse blood samples. The right panel of Figure 6(c) shows that high transgene expression is mainly observed in blood and to a much lower extent in the spleen. The transgene tissue specific expression was confirmed by comparing the amount of the human IVSI-6 *β*-globin transgenic RNA to the endogenous murine *β*-globin mRNAs isolated from different tissues, including spleen, brain, liver, lung, stomach, and kidney (left panel of Figure 6(c)). The expression of human *β*-globin transcripts in transgenic mouse tissues (right panel, black bars) is comparable to the endogenous mouse *β*-globin transcripts (left panel, grey bars), and the highest transcription of both human and murine globin mRNAs was restricted to the splenic compartment, as expected; in addition, the results shown in Figure 6(c) demonstrate that the pattern of IVSI-6 *β*-globin RNA expression is very similar to that of murine *β*-globin RNA, strongly suggesting that the tissue specific expression is maintained in the TG-*β*-IVSI-6 line analyzed. Moreover, the endogenous expression of murine *β*-like globin genes is not perturbed by the integration of the *β*-IVSI-6 transgene.

### 3.6. Hematological Parameters of TG-*β*-IVSI-6 Mice

The hematological parameters of wild-type and transgenic TG-*β*-IVSI-6 mice are reported in Table 3. In total, we analyzed 10 wild-type and 11 TG-*β*-IVSI-6 mice, 16-week-old. No significant differences were observed in total hemoglobin content between males and females. Concerning the other parameters examined, no major differences were found, despite the fact that some hematological data support the possibility that TG-*β*-IVSI-6 mice produce higher levels of RBC (red blood cells). In addition, it should be noted that RDW (red cell distribution width) is higher and that MCV (mean corpuscular volume) and MCH (mean corpuscular hemoglobin) are lower in transgenic TG-*β*-IVSI-6 mice in respect to wild-type mice.

### 3.7. TG-*β*-IVSI-6 Mice Produce Human *β*-Globin and Synthesize Mouse/Human ^mu^ **α**-Globin_2_/^hu^ **β**-Globin_2_ Hybrid Hemoglobin

**Figure 7:**
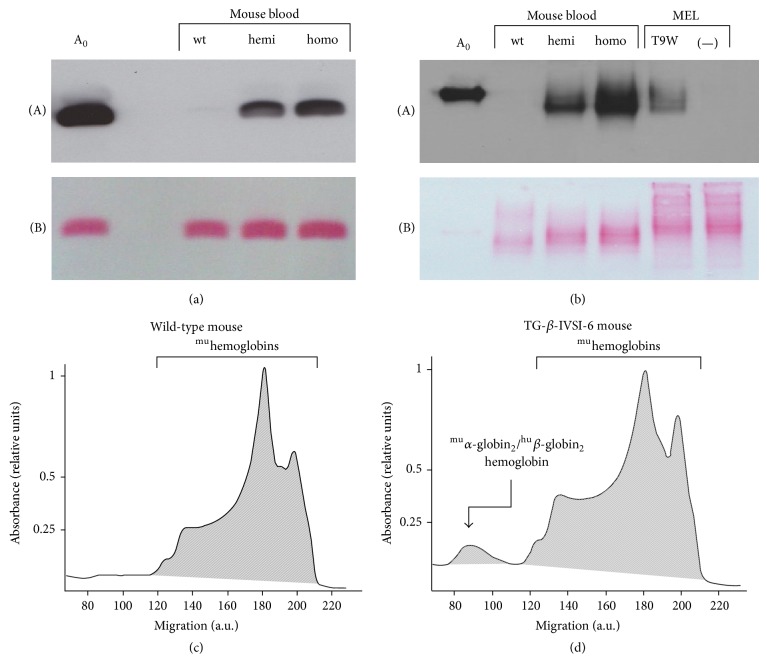
Analysis of transgenic protein synthesis in the TG-*β*-IVSI-6 mouse model. (a) Western blotting was employed to determine the presence of human *β*-globin in the blood of wild-type (wt), hemizygous (hemi), and homozygous (homo) mice by using a human *β*-globin specific primary antibody (A). Human adult A_0_ hemoglobin was used as migration reference. (b) Native western blotting was performed by using a human *β*-globin specific primary antibody (A) and by employing, as a template, wild-type (wt), hemizygous (hemi), or homozygous (homo) mouse blood, and cell extracts obtained from MEL cells either infected with T9W lentiviral vector and treated with DMSO (T9W) or treated with DMSO only (−). Red Ponceau staining was used to verify that an equal amount of sample was loaded in each well and to verify the transfer to the membrane ((B) in panels (a) and (b)). (c, d) Capillary electrophoresis of whole blood from wild-type (c) and homozygous TG-*β*-IVSI-6 (d) mice. Peaks generated by murine hemoglobins and hybrid ^mu^ *α*-globin_2_/^hu^ *β*-globin_2_ hemoglobin are indicated; a.u., arbitrary units.

We performed a western blotting experiment to determine whether the accumulated human *β*-globin mRNA is translated into human *β*-globin protein (Figure 7(a)). A human specific primary antibody was used to label the human *β*-globin. No cross-reaction with any of the endogenous murine globins was observed. A *β*-globin specific band is detectable in samples from both hemizygous and homozygous TG-*β*-IVSI-6 mice, demonstrating that the human *β*-globin mRNA produced by the transgene is translated into a normal *β*-globin. As expected, the amount of *β*-globin produced by homozygous mice is higher than that produced by hemizygous animals.

The native electrophoresis and western blotting analysis reported in Figure 7(b) suggest that a hybrid ^mu^ *α*-globin_2_/^hu^ *β*-globin_2_ hemoglobin is present in both homozygous and hemizygous TG-*β*-IVSI-6 mice. Control experiments were performed by using T9W-transduced MEL cells (see also Supplementary Figure S1 in the Supplementary Material available online at http://dx.doi.org/10.1155/2015/687635 for the analysis of the results obtained following transduction), confirming that a hybrid ^mu^ *α*-globin_2_/^hu^ *β*-globin_2_ hemoglobin can be produced when the human *β*-globin gene is expressed under a murine cellular context (Figure 7(b), right side of the panel). The qualitative western blotting shown in Figure 7(b) does not provide conclusive information about the proportion of ^mu^ *α*-globin_2_/^hu^ *β*-globin_2_ hemoglobin produced by the TG-*β*-IVSI-6 mice. Therefore, in order to estimate the percentage of hybrid ^mu^ *α*-globin_2_/^hu^ *β*-globin_2_ hemoglobin with respect to the total murine hemoglobin production, high voltage capillary electrophoresis (CE) experiments were performed (Figures 7(c) and 7(d)). This system, unlike HPLC [41], allows a clear separation between the murine Hbmajor/Hbminor and the murine/human hybrid ^mu^ *α*-globin_2_/^hu^ *β*-globin_2_ hemoglobin. The results obtained indicate that the ^mu^ *α*-globin_2_/^hu^ *β*-globin_2_ hemoglobin is clearly detectable in transgenic animals (see the representative CE analysis shown in Figure 7(d)), representing 3.9 ± 0.4% of the total hemoglobin produced in 6 TG-*β*-IVSI-6 mice analyzed.

### 3.8. Presence of Aberrantly Spliced Molecules in TG-*β*-IVSI-6 Mice

**Figure 8:**
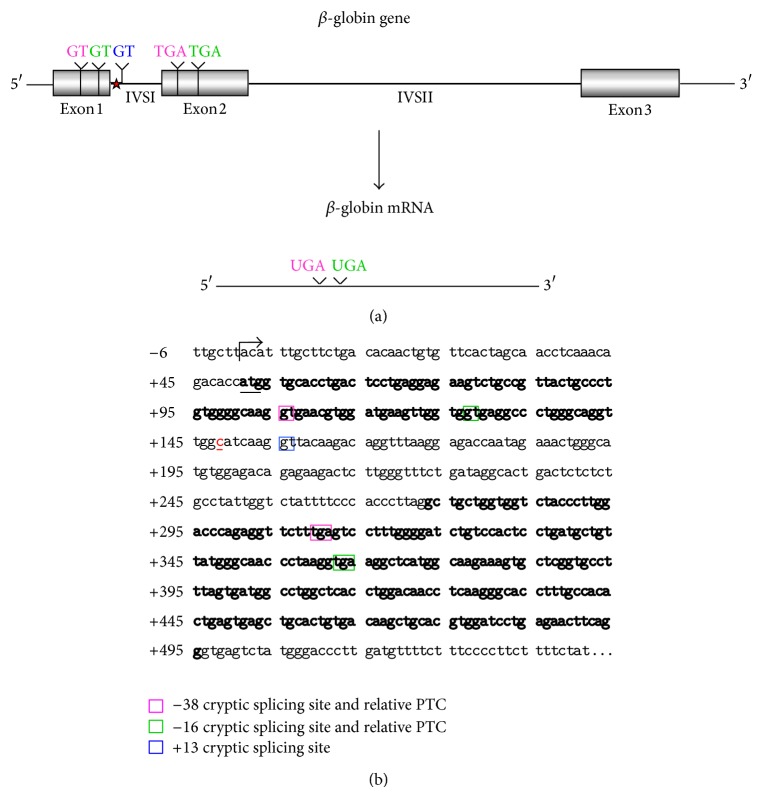
(a) Schematic representation of the human *β*-globin gene and mRNA. The three cryptic GT splicing sites activated by the IVSI-6 mutation and the two consequent stop codons are indicated with different colours. The IVSI-6 mutation (T→C) is identified by a red star. (b) Genomic region containing the first and second exons of the human *β*-globin gene, in bold characters. The IVSI-6 mutation occurring at the sixth nucleotide of the first intron is shown in red. The coloured boxes indicate the three cryptic splicing sites activated by the mutation and the two consequent stop codons. The transcription and translation starting sites are also indicated.

**Figure 9:**
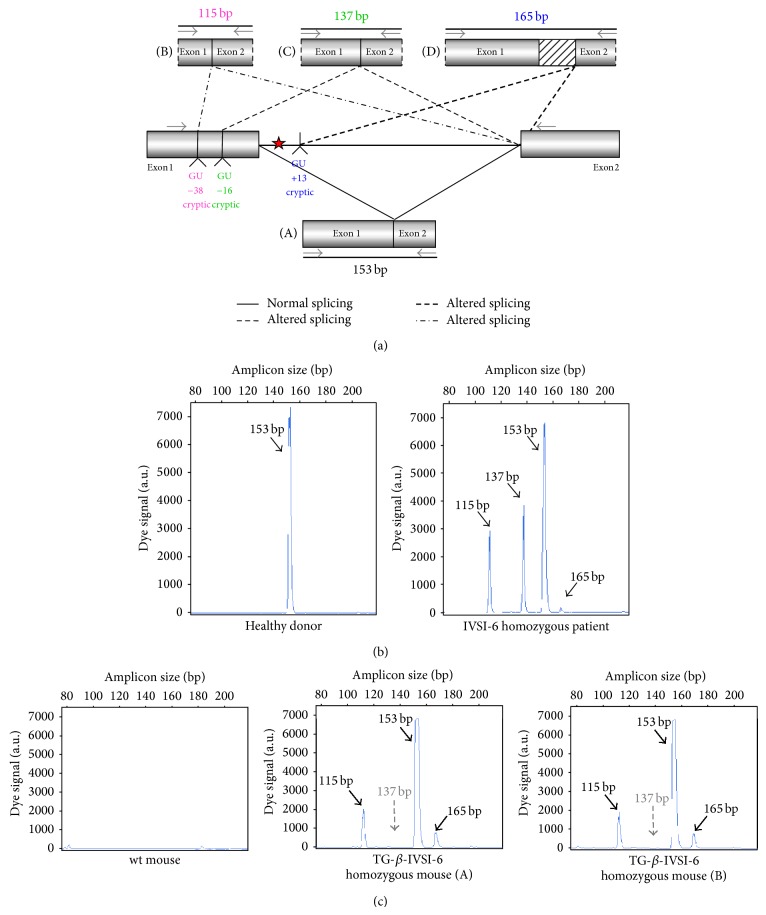
Normal and aberrant splicing in IVSI-6 *β*-globin gene. (a) Schematic representation of the normal (A) and altered splicing (B, C, D) in IVSI-6 thalassemia. Grey arrows indicate the primers used to demonstrate the presence of the altered splicing. The positions of the cryptic splicing sites generated by the mutation and the respective lengths (in bp) of products obtained after PCR amplification of altered transcripts are indicated with different colours. A red star locates the IVSI-6 mutation. (b, c) Identification of aberrantly spliced transcripts in IVSI-6 patients and in the TG-*β*-IVSI-6 mouse model. (b) Electropherograms generated by denaturing polyacrylamide gel electrophoresis of fluorescent RT-PCR products obtained from healthy donor blood (left panel) and IVSI-6 homozygous patient blood (right panel). (c) Electropherograms obtained from a wild-type and two TG-*β*-IVSI-6 mice (A, B). Primers employed were* HuBetaF1[6FAM]-HuBetaR* (Table 2). Blue peaks indicate both alternatively spliced and canonic human transcripts.

In Figure 8(a) a scheme of the mutation effects on human *β*-globin gene and mRNA is shown. The sequence containing the first and second *β*-globin gene exons is reported, and the site of the IVSI-6 mutation and the three cryptic splicing sites that may arise in IVSI-6 pre-mRNA are emphasized by coloured boxes (Figure 8(b)). A schematic representation and expected size of the normal and alternatively spliced *β*-globin transcripts in IVSI-6 thalassemic cells are shown in Figure 9(a). As expected, the electropherogram of the *β*-globin RNA, amplified from erythroid progenitor cells (ErPCs) of a healthy donor, shows only a 153 bp peak (Figure 9(b), left panel); conversely, the electropherogram of ErPCs from a homozygous IVSI-6 patient presents three additional peaks of 115, 137, and 165 bp (Figure 9(b), right panel), which represent the accumulation of the three abnormal transcripts generated by the −38, −16, and +13 cryptic GU donor splicing sites produced by the IVSI-6 point mutation, respectively. Notably, the 165 bp peak is present in lower amount, as was consistently observed in additional experiments using ErPCs from different patients (Table 4). As expected, no peak is generated by using RNA from wild-type mice (Figure 9(c), left panel), while when RNA from two TG-*β*-IVSI-6 mice is employed, both normal and abnormal transcripts are observed (Figure 9(c), middle and right panels). It should be emphasized, however, that the 137 bp peak is not present in this representative experiment, or it is present in very low amounts, as seen in additional experiments (shown in Table 4). In order to understand this issue, we used K562 cell clones stably containing the T9W-IVSI-6 vector, named K562(*β*-IVSI-6), and murine MEL cells transduced with the T9W or the T9W-IVSI-6 lentiviruses, named MEL (hu *β*-globin gene) and MEL (hu *β*-IVSI-6 globin gene), respectively. All the results obtained are shown in Table 4, demonstrating that, as expected, only the 153 bp peak is present in T9W-transduced MEL cells. Among the peaks generated by the activation of cryptic sites, the 115 bp peak is the most represented in the K562(IVSI-6) clones, as well as in MEL cells transfected with the T9W-IVSI-6 vector, while the 137 bp and 165 bp peaks are present in lower amounts. In any case the proportion of the 137 bp peak is higher than that found in TG-*β*-IVSI-6 homozygous mice. Similar patterns were observed in transduced DMSO-treated MEL cells. The different levels of transcripts (see Table 4) should be discussed by taking in consideration the hierarchy of splicing events associated with the differential extent of complementarity with U1 and U6 small nuclear RNAs (snRNAs), as suggested by Roca et al. (see also Figure 9 and Table 5) [42]. The low levels of the transcript corresponding to the 165 bp amplicon (Table 4) might be explained by the very low strength of its donor +13 cryptic splicing site, which do not generate PTCs (see Table 5). On the contrary, both the transcripts corresponding to the 115 bp and 137 bp amplicons generate PTCs, but the second one is highly unstable because of being more sensitive to nonsense mediated decay (NMD) [43, 44]. This might explain the low levels of this transcript found in TG-*β*-IVSI-6 samples, as well as in MEL cells transduced with a human *β*-IVSI-6 globin gene vector (Table 4). In any case, we like to underline that aberrant transcripts were found to be present in all the IVSI-6 experimental systems analyzed.

**Figure 10:**
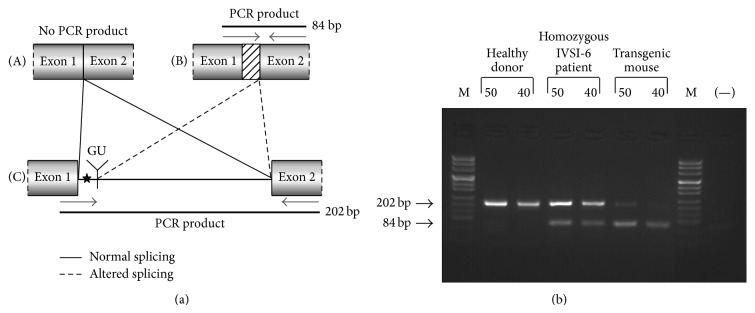
Identification of the aberrant transcripts generated by the activation of the cryptic splicing site at position IVSI+13, in the presence of IVSI-6 mutation. (a) Schematic representation of the human *β*-globin pre-mRNA spanning from the first to the second exon (C); the creation of a new aberrant donor splicing site (GU) in the first intron, the IVSI-6 mutation site (star), the location of primers used for PCR (arrows), and the PCR product lengths are indicated. Normal splicing (A) and predicted aberrant splicing (B) are also represented. (b) RT-PCR reaction was performed by using RNA from healthy subject blood, from IVSI-6 homozygous patient blood and from transgenic mouse blood. The* IVSI+13F* forward primer and the* HuBetaR* reverse primer (Table 2), designed to amplify a fragment of 84 bp from human *β*-globin altered spliced transcripts or a fragment of 202 bp from human pre-mRNA (containing all the first human *β*-globin intron), were used. The products obtained from each sample, at 40 and 50 cycles of PCR reaction, were loaded on a 3% agarose gel. M, molecular weight ladder, pUC Mix Marker 8 (Fermentas), (−), negative control.

The production of aberrant transcripts was also detected by a simple RT-PCR procedure as described in Figure 10. In this experiment, an RT-PCR reaction was performed by using RNA extracted from the ErPCs of either a healthy subject or an IVSI-6 homozygous patient and from transgenic mouse blood. For the PCR reaction the* IVSI+13F* forward primer and the* HuBetaR* reverse primer, designed to amplify a 84 bp fragment of the human *β*-globin alternatively spliced transcript or a 202 bp fragment of the human pre-mRNA (containing all the first human *β*-globin intron), were employed. A scheme of the expected PCR products is reported in Figure 10(a). The results obtained demonstrated that *β*-globin RNA precursor sequences are present in all the samples, and aberrantly spliced *β*-globin RNA sequences are present only in samples from the IVSI-6 homozygous patient and TG-*β*-IVSI-6 mouse (Figure 10(b)). Interestingly, the level of this aberrantly spliced transcript appears to be very high in the TG-*β*-IVSI-6 sample, facilitating the possible* in vivo* validation of corrections of this genetic defect.

## 4. Discussion

In this study we have reported the production and characterization of a transgenic mouse line carrying the human IVSI-6 *β*-globin gene. The IVSI-6 mutation leads to anemia associated with a *β*-thalassemia intermedia phenotype. However, the association with a *β* ^0^-like mutation (such as deletions, *β* ^0^39, and *β* ^0^IVSI-1 mutations) and even *β* ^+^ mutations renders the phenotype of the heterozygous compound more severe. Noticeably, *β* ^+^IVSI-6 thalassemia is the most common in the Middle-Eastern regions, including Egypt, Israel, Lebanon. For this reason, an* in vivo* system suitable to study possible therapeutic strategies that target the aberrantly spliced RNAs generated by this mutation is highly needed.

We generated a transgenic TG-*β*-IVSI-6 mouse, which (a) displays a tissue specific expression of the transgene, fully overlapping with that of the endogenous murine *β*-globin gene; (b) as expected it produces normally spliced human *β*-globin mRNA, giving rise to *β*-globin production and formation of a human-mouse tetrameric chimeric hemoglobin ^mu^ *α*_2_^hu^ *β*_2_ and, more importantly, (c) exhibits in blood cells aberrant IVSI-6 *β*-globin RNAs. We conclude that, despite the fact that the human *β*-IVSI-6 transgene is located in the same mouse chromosome which carries the *β*-like globin cluster (mouse chromosome 7), both the *β*-IVSI-6 transgene and the *β*-like globin cluster are expressed as expected. It should be underlined that the hematological parameters of the homozygous TG-*β*-IVSI-6 mice are very similar to those of the wild-type mice. The only significant difference we found in TG-*β*-IVSI-6 mice is the low/absent production of one aberrantly spliced transcript (the 137 bp amplicon, as shown in Figure 9). This might be explained by the fact that this particular spliced form is much more sensitive to NMD and so highly unstable (Table 5) [43, 44]. The issue of the different ratios of the transcripts corresponding to the 115, 137, 153, and 165 bp amplicons in the cellular systems considered (see Table 4) should be discussed by taking in consideration the hierarchy of splicing events associated with the differential extent of complementarity with U1 and U6 small nuclear RNAs (snRNAs), as suggested by Roca et al. (see Table 5) [42].

Despite the low stability of transcripts generated by the −16 cryptic splicing site, the presence of the other two aberrantly spliced forms (corresponding to the 115 bp and 165 bp peaks shown in Figure 9(c)) allows us to propose that the TG-*β*-IVSI-6 mouse might be used as an* in vivo* model to characterize the effects of antisense oligodeoxynucleotides (ODNs) and ODN-mimics targeting the −38 and the +13 cryptic GU donor splicing sites responsible for the generation of aberrantly spliced human *β*-globin transcripts in IVSI-6 *β*-thalassemia. The validation of the effects of molecules correcting the aberrant splicing caused by the IVSI-6 mutation can be performed* in vitro* using erythroid precursor cells isolated from these transgenic mice, as well as* in vivo* following administration of splicing correctors, as performed with different* in vitro* and* in vivo* experimental systems by several research groups [45–50].

In this respect,* ex vivo* experiments based on the correction of splicing defects causing *β*-thalassemia have been reported by several research groups using antisense phosphorothioate 2′-O-methyl-oligonucleotides [45, 46], morpholino-oligonucleotides [18, 47], 2′-O-(2-methoxy) ethyl-oligonucleotides [47], and peptide nucleic acids [48]. These antisense molecules have been used either free [45, 46] or delivered with peptides and lipid-based strategies [49]. For instance, El-Beshlawy et al. [18] reported the* ex vivo* correction of the aberrant splicing of IVSI-110 *β*-globin pre-mRNA by antisense oligonucleotides (ASONs) against the 3′ aberrant splicing site. In their study, ErPCs with the IVSI-110 mutation were treated with 20 *μ*mol/mL morpholino ASONs targeting the 3′ aberrant splicing site. The results of this work suggested that ASONs can restore correct splicing of *β*-globin pre-mRNA, leading to correct gene product.

As far as* in vivo* experiments, few reports are available [17, 50] and none of them, to the best of our knowledge, are focused on the repair of the aberrant splicing caused by the *β*-IVSI-6 mutation. For instance, Svasti et al. [17] reported the repair of defective *β*-globin pre-mRNA in a mouse model of IVSII-654 thalassemia, by delivering a morpholino oligomer conjugated to an arginine-rich peptide as splice-switching oligonucleotide (SSO). Interestingly, the SSO blocked the aberrant splicing site in the targeted pre-mRNA and forced the splicing machinery to reselect existing correct splicing sites. These results suggest the applicability of ASONs for the treatment of thalassemia.

In this respect, it is worth noting that in most of third world countries, blood transfusion is of difficult application, due to the fact that availability of blood is low and blood is often contaminated. Therefore, novel pharmacological interventions are urgently needed [51, 52].

## 5. Conclusions

Molecules able to correct the effects of *β*-IVSI-6 thalassemia mutation will be of great therapeutic interest for the *β*-thalassemia patients of the Middle-Eastern region, in which this genotype is very common. To this aim the availability of experimental systems to validate the effects of molecules protecting the activated cryptic sites (in our case the −38, the −16, and the +13 cryptic GU donor splicing sites) in the case of *β*-IVSI-6 splicing site mutations are of great interest. Suitable* in vitro* experimental system might be erythroid precursor cells from homozygous *β*-IVSI-6 patients or K562 and MEL cells carrying a *β*-IVSI-6 gene. These experimental systems, while very informative on the effects* in vitro* of splicing-regulating molecules, do not help to reach conclusive experiments* in vivo*. Our transgenic *β*-IVSI-6 experimental system, even if partially reconstituting the splicing pattern caused by the *β*-IVSI-6 mutation, might be useful to verify the* in vivo* activity of oligonucleotide-based drugs targeting the −38 GU and the +13 GU cryptic splicing sites activated in IVSI-6 *β*-thalassemia.

## Supplementary Material

Production of beta-globin mRNA by T9W-transduced MEL cells.

## Figures and Tables

**Table 1:** PCR primers employed for identification and characterization of transgenic mice.

Name	Sequence	Length (nt)	Melting temperature (°C)	Gene
*MuActF *	5′ TGACGGGGTCAACCACACTGTGCCCATCTA 3′	30	81	Murine *β*-actin
*MuActR *	5′ CTAGAAGCATTTGCGGTGGACGATGGAGGG 3′	30	80	Murine *β*-actin
*TransF *	5′ TGCATTCATTTGTTGTTGTTTTTCT 3′	25	65	Transgene (LCR)
*TransF* [*6FAM*]	5′ [6-FAM] TGCATTCATTTGTTGTTGTTTTTCT 3′	25	65	Transgene (LCR)
*TransR *	5′ TGACTAAAACTCCACCTCAAACGG 3′	24	67	Transgene (LCR)
*HuBetaF *	5′ AGACCTCACCCTGTGGAGCC 3′	20	68	Human *β*-globin
*HuBetaR *	5′ TCAGGAGTGGACAGATCCCC 3′	20	67	Human *β*-globin
*MuActF1* [*6FAM*]	5′ [6-FAM] TACTTTGGGAGTGGCAAGCC 3′	20	66	Murine *β*-actin
*MuActR1 *	5′ TCTCCATGTCGTCCCAGTTG 3′	20	66	Murine *β*-actin

**Table 2:** Primers employed for RT-PCR analyses.

Name	Sequence	Length (nt)	Melting temperature (°C)	Transcript
*HuBetaF1 *	5′ GCATCTGACTCCTGAGGAGAAGTC 3′	24	67	Human *β*-globin
*HuBetaF1* [*6FAM*]	5′ [6-FAM] GCATCTGACTCCTGAGGAGAAGTC 3′	24	67	Human *β*-globin
*HuBetaR *	5′ TCAGGAGTGGACAGATCCCC 3′	20	67	Human *β*-globin
*MuBetaF *	5′ CCTGACTGATGCTGAGAAGGC 3′	21	66	Murine *β*-globin
*MuBetaR *	5′ GCAGAGGATAGGTCTCCAAAGCTATC 3′	26	67	Murine *β*-globin
*MuAlphaF *	5′ CTGAAGCCCTGGAAAGGATGT 3′	21	66	Murine *α*-globin
*MuAlphaR *	5′ ATTTGTCCAGAGAGGCATGCA 3′	21	67	Murine *α*-globin
*MuActF2 *	5′ TGTATTCCCCTCCATCGTGG 3′	20	67	Murine *β*-actin
*MuActR2 *	5′ CACAGCCTGGATGGCTACGTAC 3′	22	68	Murine *β*-actin
*IVSI*+*13F *	5′ GGGCAGGTTGGCATCAAG 3′	18	67	IVSI+13 altered spliced transcripts

**Table 3:** Hematological data of transgenic mice carrying the human ^hu^ *β* ^IVSI-6^ globin locus.

Age (weeks)	Sex	Genotype	Hb (g/dL)	RBC (10^6^/*µ*L)	HCT (%)	MCV (fL)	MCH (pg)	MCHC (g/dL)	RDW (%)
16	♀	Wild-type (*n* = 5)	13.7 ± 0.4	8.2 ± 0.4	42.9 ± 1.8	52.5 ± 2.7	16.8 ± 0.8	31.9 ± 0.3	16.1 ± 0.9
16	♀	Homozygous ^hu^ *β* ^IVSI-6^, ^hu^ *β* ^IVSI-6^ (*n* = 5)	13.8 ± 0.2	8.8 ± 0.1	43.6 ± 1.0	49.3 ± 0.7	15.6 ± 0.2	31.7 ± 0.5	17.2 ± 0.2
			NS	*P = 0.01 *	NS	*P = 0.04 *	*P = 0.02 *	NS	*P = 0.03 *

16	♂	Wild-type (*n* = 5)	12.5 ± 0.6	7.6 ± 0.3	39.0 ± 1.4	51.5 ± 1.1	16.5 ± 0.2	31.9 ± 0.7	15.9 ± 0.8
16	♂	Homozygous ^hu^ *β* ^IVSI-6^, ^hu^ *β* ^IVSI-6^ (*n* = 6)	13.0 ± 0.3	8.6 ± 0.2	42.0 ± 1.1	49.1 ± 0.9	15.2 ± 0.1	31.0 ± 0.4	16.8 ± 0.2
			NS	*P = 0.0002 *	*P = 0.003 *	*P = 0.003 *	*P < 0.0001 *	*P = 0.03 *	*P = 0.03 *

Hematological values are expressed as means ± SD. Hemoglobin concentration (Hb), red blood cell count (RBC), hematocrit (HCT), mean corpuscular volume (MCV), mean corpuscular hemoglobin (MCH), mean corpuscular Hb concentration (MCHC), red cell distribution width (RDW) are shown. *n* indicates the number of analyzed mice. The *P*  values of  Student's *t*-test, comparing each group of transgenic mice with wild-type mice controls, are also shown. NS corresponds to not statistically significant *P* > 0.05.

**Table 4:** Relative contents of *β*-globin transcripts.

Sample	Experiment	Peaks (%)			
		115 bp	137 bp	153 bp	165 bp
Splicing site generating the transcript		−38 (cryptic)	−16 (cryptic)	+1 (normal)	+13 (cryptic)
ErPCs from normal donors	*1 *	0	0	100	0
	*2 *	0	0	100	0
	*3 *	0	0	100	0

ErPCs from homozygous *β*-IVSI-6/*β*-IVSI-6 patients	*1 *	19.9	13.6	65.8	0.7
	*2 *	16.1	13.7	69.2	1.0
	*3 *	14.4	18.7	66.2	0.7

TG-*β*-IVSI-6 homozygous mouse #1	*1 *	4.3	0.3	93.8	1.6
	*2 *	7.6	0.1	88.6	3.7

TG-*β*-IVSI-6 homozygous mouse #2	*1 *	6.5	0.4	91.1	2.0
	*2 *	5.6	0.2	92.0	2.2

K562(*β*-IVSI-6) #1	*1 *	21.6	2.2	74.6	1.6
	*2 *	21.3	2.6	74.3	1.8

K562(*β*-IVSI-6) #2	*1 *	22.3	3.0	73.6	1.1

MEL(hu *β*-globin gene)	*1 *	0	0	100	0

MEL(hu *β*-globin gene), DMSO treated	*1 *	0	0	100	0

MEL(hu *β*-IVSI-6 globin gene)	*1 *	33.1	6.8	58.7	1.4

MEL(hu *β*-IVSI-6 globin gene), DMSO treated	*1 *	38.5	5.8	54.5	1.2

**Table 5:** Strengths of the normal and cryptic splicing sites generated by the *β* ^+^IVSI-6 thalassemic mutation.

Splicing site	Sequence	Base pairs	Strength			Amplicon size	Comments/Hypotheses
			(a)	(b)	(c)		
+1 (normal)	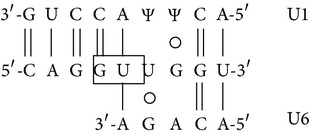	7+ 3+	86.64	8.08	0.64	153 bp	

+1 (mutated IVSI-6)	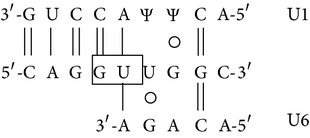	6+ 2+	84.46	5.52	0.14	153 bp	

−38 (cryptic)	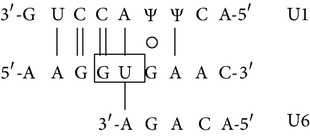	5+ 1	83.50	5.54	0.21	115 bp	PTC. Unstable transcript, low sensitivity to NMD (in comparison with the −16 cryptic splicing site)

−16 (cryptic)	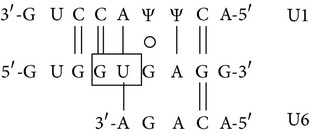	5+ 2	90.40	6.13	0.54	137 bp	PTC. Unstable transcript, sensitive to NMD

+13 (cryptic)	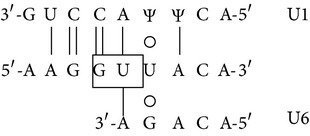	5+ 1+	79.67	−0.83	0.46	165 bp	No PTC. GT located 7 nucleotides downstream the IVSI-6 mutation

Sequences of the normal and cryptic splicing sites generated by the *β* ^+^IVSI-6 thalassemic mutation. The cryptic donor GU (boxed) sites are numbered with respect to the +1 position of the normal one. Potential Watson-Crick base pairs to U1 (upper) and U6 (lower) are quantified: (+) indicates a G/U wobble base pair. Strengths of donor splicing sites are expressed as scores calculated with Human Splicing Finder Matrices from http://www.umd.be/HSF/ (a) or MaxEntScan from http://www.umd.be/HSF/ (b) or from http://www.fruitfly.org/seq_tools/splice.html (c).

## Abbreviations

HPLC: High performance liquid chromatography
FBS: Fetal bovine serum
PBS: Phosphate-buffered saline
RBC: Red blood cell
bp: Base pairs
Kb: Kilobases
DMSO: Dimethyl sulfoxide
PCR: Polymerase chain reaction
TBS: Tris-buffered saline
LCR: Locus control region
QMPSF: Quantitative multiplex PCR of short fluorescent fragments
ErPC: Erythroid precursor cells
ODN: Oligodeoxy nucleotides
Hb: Hemoglobin
nt: Nucleotides
FISH: Fluorescence* in situ* hybridization.
