# Supplementary material for: Generation and Characterization of a Transgenic Mouse Carrying a Functional Human β-Globin Gene with the IVSI-6 Thalassemia Mutation
Source: Biomed Res Int. 2015 May 4;2015:687635. doi: 10.1155/2015/687635 (PMC4434229; doi:10.1155/2015/687635)
Supplement: Supplementary file 1 — Production of beta-globin mRNA by T9W-transduced MEL cells. [file 687635.f1.pdf]

Generation and characterization of a transgenic mouse carrying a functional human β-globin gene with the IVSI-6 thalassemia mutation

SUPPLEMENTARY MATERIALS

RESULTS

MEL cells transduced with T9W lentiviral vector produce β-globin mRNA.

We induced with dimethyl sulfoxide (DMSO) murine erythroleukemic cells (MEL), previously transduced with the T9W lentiviral vector carrying the human β-globin gene [31], to produce *in vitro* $^{mu}\alpha$-globin$_2$/$^{hu}\beta$-globin$_2$ hemoglobin, as a control. Supplementary Figure S1 shows cell proliferation (Figure S1A), erythroid differentiation (Figure S1B) and β-globin mRNA content (Figure S1C) of MEL cells under the different experimental conditions. Only a minimal toxicity is observed in undifferentiated or DMSO-treated cells transduced with T9W (Figure S1A). The transduction with T9W does not interfere with DMSO-induced differentiation (Figure S1B), and T9W alone does not induce differentiation. Panel C of Figure S1 demonstrates that untreated and DMSO-treated cells express murine β-globin mRNA exclusively (Figure S1C, white bars), although to a different extent. Interestingly, some human β-globin mRNA was found in T9W-transduced cells at baseline but to a much higher extent after DMSO induction (Figure S1C, black bars).

LEGENDS TO SUPPLEMENTARY FIGURES

Supplementary Figure S1.

Effects on MEL cells of treatment with DMSO, infection with T9W lentiviral vector or both: (A) cell proliferation, as cell number/ml; (B) benzidine assay, expressed as percentage of Hb-positive cells; (C) quantitative real-time PCR to identify the expression of human (black bars) and murine (white bars) β-globin transcripts, normalized over control.

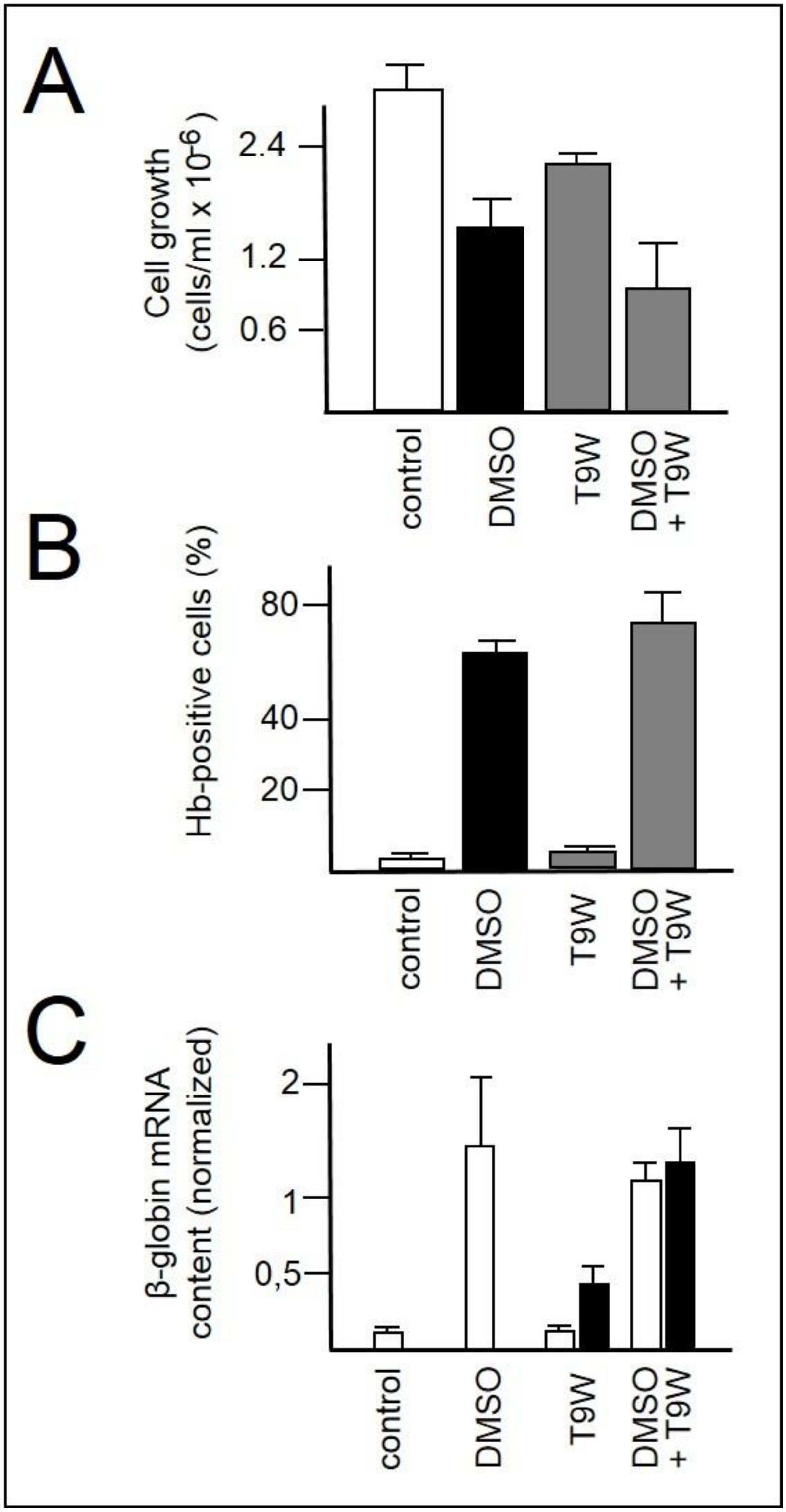

Supplementary Figure S1
